# An Analytical Technique, Based on Natural Transform to Solve Fractional-Order Parabolic Equations

**DOI:** 10.3390/e23081086

**Published:** 2021-08-21

**Authors:** Ravi P. Agarwal, Fatemah Mofarreh, Rasool Shah, Waewta Luangboon, Kamsing Nonlaopon

**Affiliations:** 1 Department of Mathematics, Texas A & M University-Kingsville, Kingsville, TX 78363, USA; ravi.agarwal@tamuk.edu; 2 Department of Mathematical Sciences College of Sciences, Princess Nourah Bint Abdulrahman University, Riyadh 11546, Saudi Arabia; fyalmofarrah@pnu.edu.sa; 3 Department of Mathematics, Abdul Wali khan University, Mardan 23200, Pakistan; rasoolshahawkum@gmail.com; 4 Department of Mathematics, Faculty of Science, Khon Kaen University, Khon Kaen 40002, Thailand; waewta_l@kkumail.com

**Keywords:** natural transform decomposition method, fractional-order parabolic equations, Caputo–Fabrizio operator

## Abstract

This research article is dedicated to solving fractional-order parabolic equations using an innovative analytical technique. The Adomian decomposition method is well supported by natural transform to establish closed form solutions for targeted problems. The procedure is simple, attractive and is preferred over other methods because it provides a closed form solution for the given problems. The solution graphs are plotted for both integer and fractional-order, which shows that the obtained results are in good contact with the exact solution of the problems. It is also observed that the solution of fractional-order problems are convergent to the solution of integer-order problem. In conclusion, the current technique is an accurate and straightforward approximate method that can be applied to solve other fractional-order partial differential equations.

## 1. Introduction

The present research work is dedicated to studying the analytical solution of fractional-order parabolic equations. In the literature, it is well recognized that a broad range of problems in physics, engineering, nuclear physics, and mathematics can be defined as unique boundary and initial value problems. A homogeneous beam’s transverse vibrations are controlled by fractional single fourth-order parabolic partial differential equations (PDEs). Such problem types occur in viscoelastic and inelastic flow mathematical modeling, layer deflection theories, and beam deformation [1,2,3,4,5,6,7,8]. Analyses of these problems have drawn the attention of several physicists and mathematicians.

The time fractional parabolic PDEs with variable coefficient:

$$\begin{array}{c} \frac{\partial^{\gamma }\upsilon }{\partial \eta^{\gamma }}+\kappa \left(\xi ,\phi ,\psi \right)\frac{\partial^{4}\upsilon }{\partial \xi^{4}}+\frac{1}{\phi }\mu \left(\xi ,\phi ,\psi \right)\frac{\partial^{4}\upsilon }{\partial \phi^{4}}+\frac{1}{\psi }\rho \left(\xi ,\phi ,\psi \right)\frac{\partial^{4}\upsilon }{\partial \psi^{4}}=g\left(\xi ,\phi ,\psi ,\eta \right),\\ 1<\gamma \leq 2,\;\eta \geq 0, \end{array}$$

where κ(ξ,ϕ,ψ), μ(ξ,ϕ,ψ) and ρ(ξ,ϕ,ψ) are positive, with initial condition

$$\begin{array}{cc} \; & \upsilon \left(\xi ,\phi ,\psi ,\eta \right)=f_{0}\left(\xi ,\phi ,\psi \right),\\ \; & \upsilon_{\eta }\left(\xi ,\phi ,\psi ,\eta \right)=k_{0}\left(\xi ,\phi ,\psi \right), \end{array}$$

with boundary conditions

$$\begin{array}{cc} \; & \upsilon \left(a,\phi ,\psi ,\eta \right)=h_{0}\left(\phi ,\psi ,\eta \right),\;\upsilon \left(b,\phi ,\psi ,\eta \right)=h_{1}\left(\phi ,\psi ,\eta \right),\\ \; & \upsilon \left(\xi ,a,\psi ,\eta \right)=g_{0}\left(\phi ,\psi ,\eta \right),\;\upsilon \left(\xi ,b,\psi ,\eta \right)=g_{1}\left(\phi ,\psi ,\eta \right),\\ \; & \upsilon \left(\xi ,\phi ,a,\eta \right)=k_{0}\left(\phi ,\psi ,\eta \right),\;\upsilon \left(\xi ,\phi ,b,\eta \right)=k_{1}\left(\phi ,\psi ,\eta \right),\\ \; & \upsilon_{\xi \xi }\left(a,\phi ,\psi ,\eta \right)=\bar{h_{0}}\left(\phi ,\psi ,\eta \right),\;\upsilon_{\xi \xi }\left(b,\phi ,\psi ,\eta \right)=\bar{h_{1}}\left(\phi ,\psi ,\eta \right),\\ \; & \upsilon_{\phi \phi }\left(\xi ,a,\psi ,\eta \right)=\bar{g_{0}}\left(\phi ,\psi ,\eta \right),\;\upsilon_{\phi \phi }\left(\xi ,b,\psi ,\eta \right)=\bar{g_{1}}\left(\phi ,\psi ,\eta \right),\\ \; & \upsilon_{\psi \psi }\left(\xi ,\phi ,a,\eta \right)=\bar{k_{0}}\left(\phi ,\psi ,\eta \right),\;\upsilon_{\psi \psi }\left(\xi ,\phi ,b,\eta \right)=\bar{k_{1}}\left(\phi ,\psi ,\eta \right). \end{array}$$

for which $h_{\ell }$, $g_{\ell }$, $k_{\ell }$, $h_{\ell }$, $g_{\ell }$, $k_{\ell }$ are continuous variables and *ℓ* differs between 0 and 1, which is the beam’s flexural stiffness ratio [1] in its volume per unit mass, as mentioned in [1,2,3,4,5,6,7]. After being used for the first time in underwater acoustics, the parabolic equation has undergone extensive development, including improvements in accuracy and implementation in the time domain. With the introduction of the wide-angle parabolic equation, the phase errors of parabolic equation solutions, which approximate the solution of the wave equation, were greatly reduced. While various generalisations of the wide-angle parabolic equation have been considered, the parabolic equation’s aperture limitation has remained a source of concern. The time-domain parabolic equation enables one to calculate pulse propagation without using Fourier synthesis. The time-domain parabolic equation has been expanded to account for interface conditions, nonlinear propagation, density variations and sediment attenuation, as well as wide-angle diffraction and sediment dispersion. Many researchers [9,10] have attempted to study the analytical solutions of parabolic equations of the fourth order. Different techniques have been suggested recently, such as the B-spline method [11], the decomposition method [12], the implicit scheme [13] and the Spline method [14], to analyze the solution of the partial differential fourth-order parabolic equation. Biazar and Ghazvini [15] used He’s iterative technique for the solution of parabolic PDEs. The modified version of this method was introduced in [16] to solve singular fourth-order parabolic PDEs. The fourth-order parabolic PDE analytical solution was examined in [17]. The modified Laplace variational iteration technique was discussed by [18] to solve singular fourth-order parabolic PDEs.

Rawashdeh and Maitama developed a new method, which was named the natural transform decomposition method (NTDM) in 2014, to handle linear and non-linear PDEs and ODEs that occur in several applications of mathematical engineering and physics [19]. NDM is a combination of NTM [20] ADM [21]. The suggested method provides a series from a solution which converges quickly to an exact solution in a closed form, see Belgacem and Silambarasan [22]. The NTDM provides analytical results of fractional-order heat and wave problems [23]. The NTDM provides an analytical solution by using fractional-order delay PDEs [24]. Different linear and non-linear PDEs and ODEs, such as fractional diffusion equations, are solve by using NTDM [25], fractional non-linear systems of PDEs [26], fractional telegraph equation [27], and time-fractional coupled Burger equations [28].

## 2. Preliminaries

**Definition** **1.** *The following transformation of $\bar{f}\left(\eta \right)$ is called natural transformation and is expressed as [29,30]*

$$N^{+}\left[\bar{f}\left(\eta \right)\right]=Q_{1}\left(s,u\right)=\frac{1}{u}\int_{0}^{\infty }e^{\frac{-s\eta }{u}}\bar{f}\left(\eta \right)d\eta ;\;s,u>0,$$

*where the transformation parameters are s and u.*

**Definition** **2.** *The following transformation of $\bar{f}\left(\eta \right)$ is called inverse natural transformation and is expressed as*

$$N^{-}\left[Q_{1}\left(s,u\right)\right]=\bar{f}\left(\eta \right)=\frac{1}{2\pi i}\int_{p_{1}-i\infty }^{p_{1}+i\infty }e^{\frac{s\eta }{u}}Q_{1}\left(s,u\right)ds,$$

*where s and u denote the transformation factors and $s=p_{1}$ in the complex plane $s=\xi_{1}+i\phi_{1}$ is taken as an integral.*

**Definition** **3.** *The nth derivative in term of NT*

*The nth derivative in term of NT ${\bar{f}}^{\ell }\left(\eta \right)$ is the $\bar{f}\left(\eta \right)$ and is defined as*

$$N\left[{\bar{f}}^{\ell }\left(\eta \right)\right]=Q_{\ell }\left(s,u\right)=\frac{s^{\ell }}{u^{\ell }}Q_{1}\left(s,u\right)-\sum_{k=0}^{\ell -1}\frac{s^{\ell -(k+1)}}{u^{\ell -k}}{\bar{f}}^{k}\left(0\right),\;\ell \geq 1.$$

**Theorem** **1.** *If $H_{1}\left(s,u\right)$, $L_{1}\left(s,u\right)$ is the natural transformation of the corresponding functions $h_{1}\left(\eta \right)$ and $l_{1}\left(\eta \right)$ are both set to A, then $H_{1}\left(s,u\right)$ is the natural transformation*

$$N\left[h_{1}*l_{1}\right]=uH_{1}\left(s,u\right)L_{1}\left(s,u\right),$$

*a where $h_{1}*l_{1}$ represents the convolution of $h_{1}$ and $l_{1}$.*

**Definition** **4.** *The Riemann–Liouville fractional-order integral [31,32]*

$$I_{\xi }^{\gamma }\bar{f}\left(\xi \right)=\left\{\begin{array}{cc} \bar{f}\left(\xi \right) & \mathrm{if}\;\gamma =0,\\ \frac{1}{\Gamma (\gamma )}\int_{0}^{\xi }{\left(\xi -\upsilon \right)}^{\gamma -1}\bar{f}\left(\upsilon \right)d\upsilon & \mathrm{if}\;\gamma >0, \end{array}\right.$$

*where* Γ *is a function defined by*

$$\Gamma \left(\Gamma \right)=\int_{0}^{\infty }e^{-\xi }\xi^{\omega -1}d\xi \;\omega \in C.$$

**Definition** **5.** *The Caputo fractional derivative operator with order γ is defined as [33]*

$$D^{\gamma }\bar{f}\left(\xi \right)=\frac{\partial^{\gamma }\bar{f}\left(\xi \right)}{\partial \eta^{\gamma }}=\left\{\begin{array}{cc} I^{\ell -\gamma }\left[\frac{\partial^{\gamma }\bar{f}\left(\xi \right)}{\partial \eta^{\gamma }}\right], & \mathrm{if}\;\ell -1<\gamma \leq \ell ,\;\ell \in N.\\ \frac{\partial^{\gamma }\bar{f}\left(\xi \right)}{\partial \eta^{\gamma }}. \end{array}\right.$$

*where ℓ∈N, ξ>0, $\bar{f}\in C_{\eta }$, η≥−1.*

**Definition** **6.** *Natural transform of $D_{\eta }^{\gamma }u\left(\eta \right)$ by means of Caputo–Fabrizio is defined as*

(1) $$\begin{array}{c} N\left[D_{\eta }^{\gamma }\right]=\frac{1}{1-\gamma +\gamma (\frac{u}{s})}\left(N\left[\upsilon \left(\eta \right)\right]-\left(\frac{1}{s}\right)\upsilon \left(0\right)\right). \end{array}$$

## 3. Idea of FNTM

The general fractional-order PDEs are given as

(2) $${}^{CF}D^{\gamma }\upsilon \left(\xi ,\eta \right)+L\upsilon \left(\xi ,\eta \right)+N\upsilon \left(\xi ,\eta \right)=q\left(\xi ,\eta \right),\;\xi ,\eta \geq 0,\;m-1<\gamma <m,$$

where $D^{\gamma }=\frac{\partial^{\gamma }}{\partial \eta^{\gamma }}$ represents the fractional derivative in term of Caputo sense. Moreover, *L* is the linear and *N* is the non-linear term in Equation (1).

The initial condition is

(3) υ(ξ,0)=k(ξ),0<γ≤1,η>0.

Applying the natural transformation to Equation (1), we get

(4) $$N^{+}\left[D^{\gamma }\upsilon \left(\xi ,\eta \right)\right]+N^{+}\left[L\upsilon (\xi ,\eta )+N\upsilon (\xi ,\eta )\right]=N^{+}\left[q(\xi ,\eta )\right],$$

Using the natural transform’s differentiation property, we get

$$N^{+}\left[\upsilon (\xi ,\eta )\right]=\frac{1}{s}\upsilon \left(\xi ,0\right)+\frac{u(s-\gamma (s-u))}{s^{4}}N^{+}\left[q(\xi ,\eta )\right]-\frac{u(s-\gamma (s-u))}{s^{4}}N^{+}\left[L\upsilon (\xi ,\eta )+N\upsilon (\xi ,\eta )\right].$$

Now υ(ξ,0)=k(ξ)

(5) $$N^{+}\left[\upsilon (\xi ,\eta )\right]=\frac{k(\xi )}{s}+\frac{u(s-\gamma (s-u))}{s^{4}}N^{+}\left[q(\xi ,\eta )\right]-\frac{u(s-\gamma (s-u))}{s^{4}}N^{+}\left[L\upsilon (\xi ,\eta )+N\upsilon (\xi ,\eta )\right].$$

The following infinite series represents the NTDM solution υ(ξ,η)

(6) $$\upsilon \left(\xi ,\eta \right)=\sum_{j=0}^{\infty }\upsilon_{j}\left(\xi ,\eta \right),$$

and Adomian polynomials as

(7) $$N\upsilon (\xi ,\eta )=\sum_{j=0}^{\infty }A_{j},$$

(8) $$A_{j}=\frac{1}{j!}{\left[\frac{d^{j}}{d\lambda^{j}}\left[N\sum_{j=0}^{\infty }\left(\lambda^{j}\upsilon_{j}\right)\right]\right]}_{\lambda =0},\;j=0,1,2\cdots$$

We get replacement Equations (5) and (6) in Equation (4).

(9) $$N^{+}\left[\sum_{j=0}^{\infty }\upsilon_{j}\left(\xi ,\eta \right)\right]=\frac{k(\xi )}{s}+\frac{u(s-\gamma (s-u))}{s^{4}}N^{+}\left[q(\xi ,\eta )\right]-\frac{u(s-\gamma (s-u))}{s^{4}}N^{+}\left[L\sum_{j=0}^{\infty }\upsilon_{j}\left(\xi ,\eta \right)+\sum_{j=0}^{\infty }A_{j}\right].$$

Applying the natural transformation’s linearity,

$$N^{+}\left[\upsilon_{0}\left(\xi ,\eta \right)\right]=\frac{k(\xi )}{s}+\frac{u(s-\gamma (s-u))}{s^{4}}N^{+}\left[q(\xi ,\eta )\right],\;\;\;\;\;\;\;\;\;\;\;\;\left(8a\right)$$

$$N^{+}\left[\upsilon_{1}\left(\xi ,\eta \right)\right]=-\frac{u(s-\gamma (s-u))}{s^{4}}N^{+}\left[L\upsilon_{0}\left(\xi ,\eta \right)+A_{0}\right].$$

We can generally write

(10) $$N^{+}\left[\upsilon_{j+1}\left(\xi ,\eta \right)\right]=-\frac{u(s-\gamma (s-u))}{s^{4}}N^{+}\left[L\upsilon_{j}\left(\xi ,\eta \right)+A_{j}\right],\;j\geq 1.$$

Equations (9) and (10) implementing the inverse natural transformation

$$\upsilon_{0}\left(\xi ,\eta \right)=k\left(\xi \right)+N^{-}\left[\frac{u(s-\gamma (s-u))}{s^{4}}N^{+}\left[q(\xi ,\eta )\right]\right],$$

(11) $$\upsilon_{j+1}\left(\xi ,\eta \right)=-N^{-}\left[\frac{u(s-\gamma (s-u))}{s^{4}}N^{+}\left[L\upsilon_{j}\left(\xi ,\eta \right)+A_{j}\right]\right].$$

## 4. Numerical Implementation

### 4.1. Problem

Consider fractional-order one-dimensional parabolic equation:

(12) $$\frac{\partial^{\gamma +1}\upsilon }{\partial \eta^{\gamma +1}}+\left(\frac{1}{\xi }+\frac{\xi^{4}}{120}\right)\frac{\partial^{4}\upsilon }{\partial \xi^{4}}=0,\;0<\gamma \leq 1,\;\eta \geq 0,$$

with initial condition

(13) $$\begin{array}{cc} \; & \upsilon \left(\xi ,0\right)=0,\;\;\upsilon_{\eta }\left(\xi ,0\right)=1+\frac{\xi^{5}}{120}, \end{array}$$

with boundary conditions

(14) $$\begin{array}{cc} \; & \upsilon \left(\frac{1}{2},\eta \right)=\left(1+\frac{{\left(\frac{1}{2}\right)}^{5}}{120}\right)\sin\left(\eta \right),\;\upsilon \left(1,\eta \right)=\frac{121}{120}\sin\left(\eta \right),\\ \; & \frac{\partial^{2}\upsilon }{\partial \xi^{2}}\left(\frac{1}{2},\eta \right)=\frac{1}{6}{\left(\frac{1}{2}\right)}^{3}\sin\left(\eta \right),\;\frac{\partial^{2}\upsilon }{\partial \xi^{2}}\left(1,\eta \right)=\frac{1}{6}\sin\left(\eta \right). \end{array}$$

Concerning the natural transformation of (12), we get

$$\upsilon (\xi ,s,u)=\frac{1}{s}\left(0\right)+\frac{u}{s^{2}}\left(1+\frac{\xi^{5}}{120}\right)-\frac{u(s-\gamma (s-u))}{s^{4}}N^{+}\left[\left(\frac{1}{\xi }+\frac{\xi^{4}}{120}\right)\frac{\partial^{4}\upsilon }{\partial \xi^{4}}\right].$$

Using the inverse natural transformation,

$$\upsilon \left(\xi ,\eta \right)=N^{-}\left[\frac{u}{s^{2}}\left(1+\frac{\xi^{5}}{120}\right)-\frac{u(s-\gamma (s-u))}{s^{4}}N^{+}\left[\left(\frac{1}{\xi }+\frac{\xi^{4}}{120}\right)\frac{\partial^{4}\upsilon }{\partial \xi^{4}}\right]\right],$$

(15) $$\upsilon \left(\xi ,\eta \right)=\left(1+\frac{\xi^{5}}{120}\right)\eta -N^{-}\left[\frac{u(s-\gamma (s-u))}{s^{4}}N^{+}\left[\left(\frac{1}{\xi }+\frac{\xi^{4}}{120}\right)\frac{\partial^{4}\upsilon }{\partial \xi^{4}}\right]\right].$$

The Equation (15) correction function is provided by

(16) $$\sum_{\ell =0}^{\infty }\upsilon_{\ell +1}\left(\xi ,\eta \right)=\left(1+\frac{\xi^{5}}{120}\right)\eta -N^{-}\left[\frac{u(s-\gamma (s-u))}{s^{4}}N^{+}\left[\left(\frac{1}{\xi }+\frac{\xi^{4}}{120}\right)\sum_{\ell =0}^{\infty }\frac{\partial^{4}\upsilon_{\ell }}{\partial \xi^{4}}\right]\right],$$

The first term

(17) $$\upsilon_{0}\left(\xi ,\eta \right)=\left(1+\frac{\xi^{5}}{120}\right)\eta ,$$

Then we got

(18) $$\upsilon_{\ell +1}\left(\xi ,\eta \right)=-N^{-}\left[\frac{u(s-\gamma (s-u))}{s^{4}}N^{+}\left[\left(\frac{1}{\xi }+\frac{\xi^{4}}{120}\right)\sum_{\ell =0}^{\infty }\frac{\partial^{4}\upsilon_{\ell }}{\partial \xi^{4}}\right]\right],$$

for j=0

(19) $$\begin{array}{cc} \; & \upsilon_{1}\left(\xi ,\eta \right)=-N^{-}\left[\frac{u(s-\gamma (s-u))}{s^{4}}N^{+}\left[\left(\frac{1}{\xi }+\frac{\xi^{4}}{120}\right)\frac{\partial^{4}\upsilon_{0}}{\partial \xi^{4}}\right]\right],\\ \; & \upsilon_{1}\left(\xi ,\eta \right)=-\left(1+\frac{\xi^{5}}{120}\right)\frac{\eta^{2}}{3!}\left(3-3\gamma +\gamma \eta \right). \end{array}$$

The following terms are

(20) $$\begin{array}{cc} \; & \upsilon_{2}\left(\xi ,\eta \right)=-N^{-}\left[\frac{u(s-\gamma (s-u))}{s^{4}}N^{+}\left[\left(\frac{1}{\xi }+\frac{\xi^{4}}{120}\right)\frac{\partial^{4}\upsilon_{1}}{\partial \xi^{4}}\right]\right]=\left(1+\frac{\xi^{5}}{120}\right)\frac{\eta^{4}}{5!}\left(\gamma \eta +5-5\gamma \right),\\ \; & \upsilon_{3}\left(\xi ,\eta \right)=-N^{-}\left[\frac{u(s-\gamma (s-u))}{s^{4}}N^{+}\left[\left(\frac{1}{\xi }+\frac{\xi^{4}}{120}\right)\frac{\partial^{4}\upsilon_{2}}{\partial \xi^{4}}\right]\right]=-\left(1+\frac{\xi^{5}}{120}\right)\frac{\eta^{6}}{7!}\left(\gamma \eta +7-7\gamma \right)\cdots , \end{array}$$

The series form the solution of Problems (4.1), such as:

$$\upsilon \left(\xi ,\eta \right)=\upsilon_{0}\left(\xi ,\eta \right)+\upsilon_{1}\left(\xi ,\eta \right)+\upsilon_{2}\left(\xi ,\eta \right)+\upsilon_{3}\left(\xi ,\eta \right)+\upsilon_{4}\left(\xi ,\eta \right)\cdots .$$

$$\upsilon \left(\xi ,\eta \right)=\left(1+\frac{\xi^{5}}{120}\right)\left\{\eta -\frac{\eta^{2}}{3!}\left(3-3\gamma +\gamma \eta \right)+\frac{\eta^{4}}{5!}\left(\gamma \eta +5-5\gamma \right)-\frac{\eta^{6}}{7!}\left(\gamma \eta +7-7\gamma \right)+\cdots \right\},$$

When γ=1, the integer NDM solution is

(21) $$\upsilon \left(\xi ,\eta \right)=\left(1+\frac{\xi^{5}}{120}\right)\left\{\eta -\frac{\eta^{3}}{3!}+\frac{\eta^{5}}{5!}-\frac{\eta^{7}}{7!}+\frac{\eta^{9}}{9!}\cdots \right\}.$$

The exact solution is

$$\upsilon \left(\xi ,\eta \right)=\left(1+\frac{\xi^{5}}{120}\right)\sin\left(\eta \right).$$

**Figure 1:**
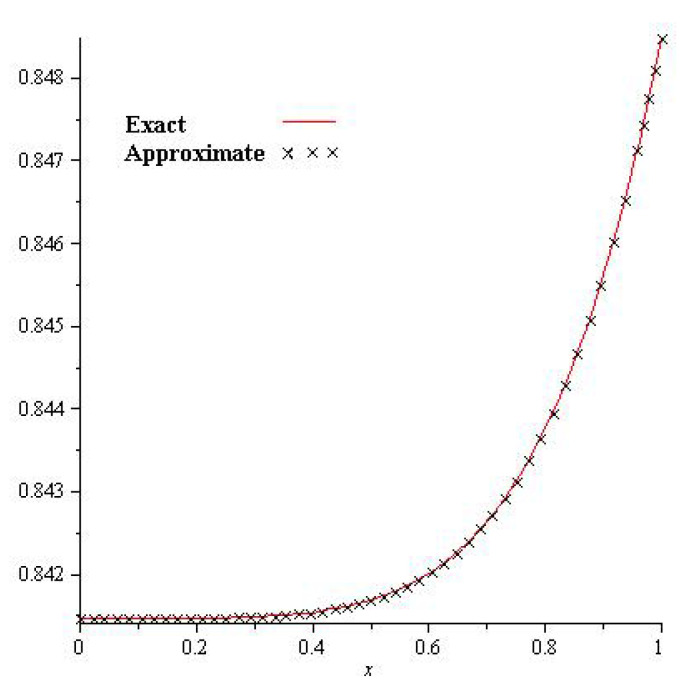
Exact and NTDM solution for γ=1 of Problem 4.1.

**Figure 2:**
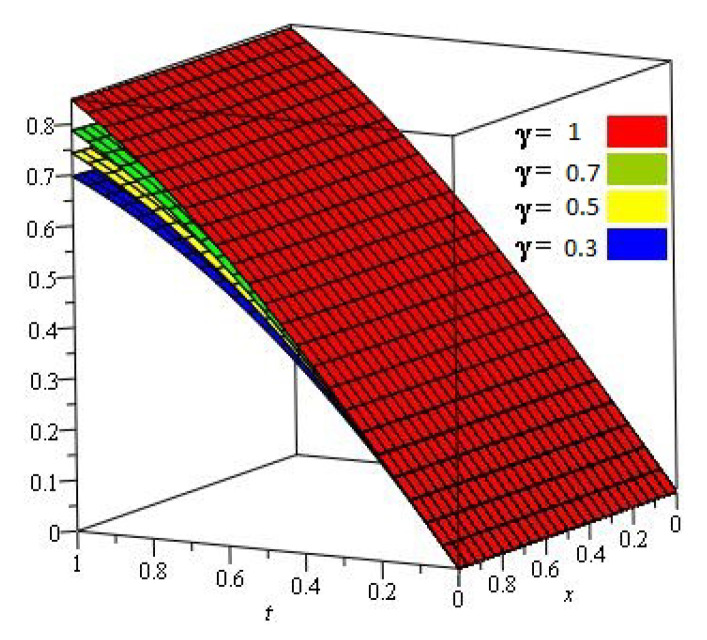
For different values of γ of Problem 4.1.

Figure 1, show that the exact and analytical solution graph of Problem 4.1. In Figure 2, the obtained solutions of Problem 4.1 are plotted at various fractional orders of the derivatives; it is confirmed that the exact and derived results are in close contact with each other. Thus the proposed method provided an accurate solution for Problem 4.1.

### 4.2. Problem

Consider fractional-order two-dimensional parabolic equation:

(22) $$\frac{\partial^{\gamma +1}\upsilon }{\partial \eta^{\gamma +1}}+2\left(\frac{1}{\xi^{2}}+\frac{\xi^{4}}{6!}\right)\frac{\partial^{4}\upsilon }{\partial \xi^{4}}+2\left(\frac{1}{\phi^{2}}+\frac{\phi^{4}}{6!}\right)\frac{\partial^{4}\upsilon }{\partial \phi^{4}}=0,\;0<\gamma \leq 1,\;\eta \geq 0,$$

with initial condition

(23) $$\begin{array}{cc} \; & \upsilon \left(\xi ,\phi ,0\right)=0,\;\;\upsilon_{\eta }\left(\xi ,\phi ,0\right)=2+\frac{\xi^{6}}{6!}+\frac{\phi^{6}}{6!}, \end{array}$$

with boundary conditions

(24) $$\begin{array}{cc} \; & \upsilon \left(\frac{1}{2},\phi ,\eta \right)=\left(2+\frac{{\left(\frac{1}{2}\right)}^{6}}{6!}+\frac{\phi^{6}}{6!}\right)\sin\left(\eta \right),\;\upsilon \left(\frac{1}{2},\phi ,\eta \right)=\left(2+\frac{{\left(1\right)}^{6}}{6!}+\frac{\phi^{6}}{6!}\right)\sin\left(\eta \right),\\ \; & \upsilon_{\xi \xi }\left(\frac{1}{2},\phi ,\eta \right)=\left(\frac{{\left(\frac{1}{2}\right)}^{4}}{4!}\right)\sin\left(\eta \right),\;\upsilon_{\xi \xi }\left(\frac{1}{2},\phi ,\eta \right)=\frac{1}{24}\sin\left(\eta \right),\\ \; & \upsilon_{\phi \phi }\left(\xi ,\frac{1}{2},\eta \right)=\frac{{\left(\frac{1}{2}\right)}^{4}}{4!}\sin\left(\eta \right),\;\upsilon_{\phi \phi }\left(\xi ,\frac{1}{2},\eta \right)=\frac{1}{24}\sin\left(\eta \right). \end{array}$$

Concerning the natural transformation of (22), we get

$$\upsilon (\xi ,\phi ,s,u)=\frac{1}{s}\left(0\right)+\frac{u}{s^{2}}\left(2+\frac{\xi^{6}}{6!}+\frac{\phi^{6}}{6!}\right)-\frac{u(s-\gamma (s-u))}{s^{4}}N^{+}\left[2\left(\frac{1}{\xi^{2}}+\frac{\xi^{4}}{6!}\right)\frac{\partial^{4}\upsilon }{\partial \xi^{4}}+2\left(\frac{1}{\phi^{2}}+\frac{\phi^{4}}{6!}\right)\frac{\partial^{4}\upsilon }{\partial \phi^{4}}\right],$$

using inverse natural transformation.

$$\upsilon \left(\xi ,\phi ,\eta \right)=N^{-}\left[\frac{u}{s^{2}}\left(2+\frac{\xi^{6}}{6!}+\frac{\phi^{6}}{6!}\right)-\frac{u(s-\gamma (s-u))}{s^{4}}N^{+}\left\{2\left(\frac{1}{\xi^{2}}+\frac{\xi^{4}}{6!}\right)\frac{\partial^{4}\upsilon }{\partial \xi^{4}}+2\left(\frac{1}{\phi^{2}}+\frac{\phi^{4}}{6!}\right)\frac{\partial^{4}\upsilon }{\partial \phi^{4}}\right\}\right],$$

(25) $$\upsilon \left(\xi ,\phi ,\eta \right)=\left(2+\frac{\xi^{6}}{6!}+\frac{\phi^{6}}{6!}\right)\eta -N^{-}\left[\frac{u(s-\gamma (s-u))}{s^{4}}N^{+}\left\{2\left(\frac{1}{\xi^{2}}+\frac{\xi^{4}}{6!}\right)\frac{\partial^{4}\upsilon }{\partial \xi^{4}}+2\left(\frac{1}{\phi^{2}}+\frac{\phi^{4}}{6!}\right)\frac{\partial^{4}\upsilon }{\partial \phi^{4}}\right\}\right.$$

The Equation (25) correction function is provided by

(26) $$\sum_{\ell =0}^{\infty }\upsilon_{\ell +1}\left(\xi ,\phi ,\eta \right)=\left(2+\frac{\xi^{6}}{6!}+\frac{\phi^{6}}{6!}\right)\eta -N^{-}\left[\frac{u(s-\gamma (s-u))}{s^{4}}N^{+}\left\{2\left(\frac{1}{\xi^{2}}+\frac{\xi^{4}}{6!}\right)\sum_{\ell =0}^{\infty }\frac{\partial^{4}\upsilon_{\ell }}{\partial \xi^{4}}+2\left(\frac{1}{\phi^{2}}+\frac{\phi^{4}}{6!}\right)\sum_{\ell =0}^{\infty }\frac{\partial^{4}\upsilon_{\ell }}{\partial \phi^{4}}\right\}\right],$$

The first term being

(27) $$\upsilon_{0}\left(\xi ,\phi ,\eta \right)=\left(2+\frac{\xi^{6}}{6!}+\frac{\phi^{6}}{6!}\right)\eta ,$$

Then we get

(28) $$\upsilon_{\ell +1}\left(\xi ,\phi ,\eta \right)=-N^{-}\left[\frac{u(s-\gamma (s-u))}{s^{4}}N^{+}\left\{2\left(\frac{1}{\xi^{2}}+\frac{\xi^{4}}{6!}\right)\sum_{\ell =0}^{\infty }\frac{\partial^{4}\upsilon_{\ell }}{\partial \xi^{4}}+2\left(\frac{1}{\phi^{2}}+\frac{\phi^{4}}{6!}\right)\sum_{\ell =0}^{\infty }\frac{\partial^{4}\upsilon_{\ell }}{\partial \phi^{4}}\right\}\right],$$

for j=0

(29) $$\begin{array}{cc} \; & \upsilon_{1}\left(\xi ,\phi ,\eta \right)=-N^{-}\left[\frac{u(s-\gamma (s-u))}{s^{4}}N^{+}\left\{2\left(\frac{1}{\xi^{2}}+\frac{\xi^{4}}{6!}\right)\frac{\partial^{4}\upsilon_{0}}{\partial \xi^{4}}+2\left(\frac{1}{\phi^{2}}+\frac{\phi^{4}}{6!}\right)\frac{\partial^{4}\upsilon_{0}}{\partial \phi^{4}}\right\}\right],\\ \; & \upsilon_{1}\left(\xi ,\eta \right)=-\left(2+\frac{\xi^{6}}{6!}+\frac{\phi^{6}}{6!}\right)\frac{\eta^{2}}{3!}\left(3-3\gamma +\gamma \eta \right). \end{array}$$

The following terms are

(30) $$\begin{array}{cc} \; & \upsilon_{2}\left(\xi ,\phi ,\eta \right)=-N^{-}\left[\frac{u(s-\gamma (s-u))}{s^{4}}N^{+}\left\{2\left(\frac{1}{\xi^{2}}+\frac{\xi^{4}}{6!}\right)\frac{\partial^{4}\upsilon_{1}}{\partial \xi^{4}}+2\left(\frac{1}{\phi^{2}}+\frac{\phi^{4}}{6!}\right)\frac{\partial^{4}\upsilon_{1}}{\partial \phi^{4}}\right\}\right],\\ \; & \upsilon_{2}\left(\xi ,\phi ,\eta \right)=\left(2+\frac{\xi^{6}}{6!}+\frac{\phi^{6}}{6!}\right)\frac{\eta^{4}}{5!}\left(\gamma \eta +5-5\gamma \right)\\ \; & \upsilon_{3}\left(\xi ,\phi ,\eta \right)=-N^{-}\left[\frac{u(s-\gamma (s-u))}{s^{4}}N^{+}\left\{2\left(\frac{1}{\xi^{2}}+\frac{\xi^{4}}{6!}\right)\frac{\partial^{4}\upsilon_{2}}{\partial \xi^{4}}+2\left(\frac{1}{\phi^{2}}+\frac{\phi^{4}}{6!}\right)\frac{\partial^{4}\upsilon_{2}}{\partial \phi^{4}}\right\}\right],\\ \; & \upsilon_{3}\left(\xi ,\phi ,\eta \right)=-\left(2+\frac{\xi^{6}}{6!}+\frac{\phi^{6}}{6!}\right)\frac{\eta^{6}}{7!}\left(\gamma \eta +7-7\gamma \right)\cdots , \end{array}$$

The series forms a solution to Problems (4.2), for example,

$$\upsilon \left(\xi ,\phi ,\eta \right)=\upsilon_{0}\left(\xi ,\phi ,\eta \right)+\upsilon_{1}\left(\xi ,\phi ,\eta \right)+\upsilon_{2}\left(\xi ,\phi ,\eta \right)+\upsilon_{3}\left(\xi ,\phi ,\eta \right)+\upsilon_{4}\left(\xi ,\phi ,\eta \right)\cdots .$$

$$\upsilon \left(\xi ,\phi ,\eta \right)=\left(2+\frac{\xi^{6}}{6!}+\frac{\phi^{6}}{6!}\right)\left\{\eta -\frac{\eta^{2}}{3!}\left(3-3\gamma +\gamma \eta \right)+\frac{\eta^{4}}{5!}\left(\gamma \eta +5-5\gamma \right)-\frac{\eta^{6}}{7!}\left(\gamma \eta +7-7\gamma \right)+\cdots \right\}.$$

Then γ=1; the integer NDM results as

(31) $$\upsilon \left(\xi ,\phi ,\eta \right)=\left(2+\frac{\xi^{6}}{6!}+\frac{\phi^{6}}{6!}\right)\left\{\eta -\frac{\eta^{3}}{3!}+\frac{\eta^{5}}{5!}-\frac{\eta^{7}}{7!}+\frac{\eta^{9}}{9!}\cdots \right\},$$

The exact solution is

$$\upsilon \left(\xi ,\phi ,\eta \right)=\left(2+\frac{\xi^{6}}{6!}+\frac{\phi^{6}}{6!}\right)\sin\left(\eta \right).$$

**Figure 3:**
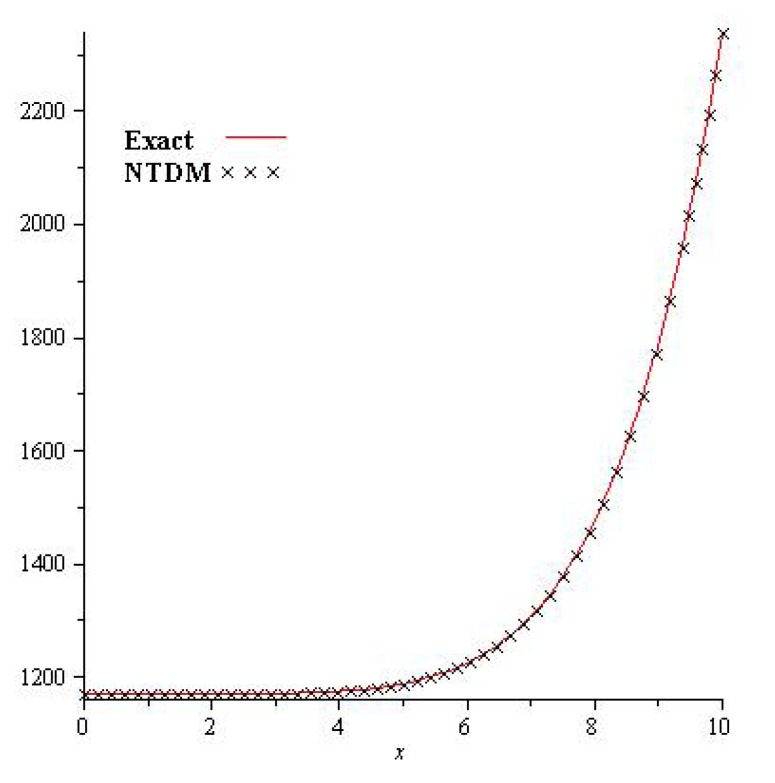
The exact and NTDM solution for γ=1 of Problem 4.2.

**Figure 4:**
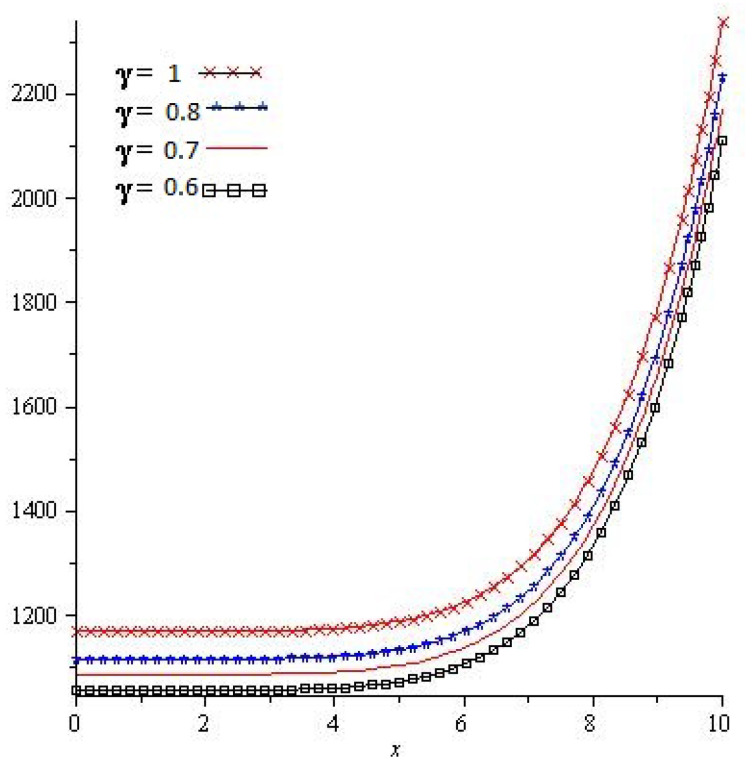
For different value of γ of Problem 4.2.

Figure 3 shows the exact and analytical solution grpah of Problem 4.2. In Figure 4, the obtained solutions of Problem 4.2 are plotted at various fractional orders of the derivatives; it is confirmed that the exact and derived results are in close contact with each other. Thus the proposed method provided an accurate solution for Problem 4.2.

### 4.3. Problem

Consider fractional-order three-dimensional parabolic equation:

(32) $$\begin{array}{cc} \; & \frac{\partial^{\gamma +1}\upsilon }{\partial \eta^{\gamma +1}}+2\left(\frac{\phi +\psi }{2\cos\xi }-1\right)\frac{\partial^{4}\upsilon }{\partial \xi^{4}}+2\left(\frac{\xi +\psi }{2\cos\phi }-1\right)\frac{\partial^{4}\upsilon }{\partial \phi^{4}}+2\left(\frac{\phi +\xi }{2\cos\psi }-1\right)\frac{\partial^{4}\upsilon }{\partial \psi^{4}}=0,\\ \; & 0<\gamma \leq 1,\;\eta \geq 0, \end{array}$$

with initial condition

(33) $$\begin{array}{cc} \; & \upsilon \left(\xi ,\phi ,\psi ,0\right)=\xi +\phi +\psi -\left(\cos(\xi )+\cos(\phi )+\cos(\psi )\right),\\ \; & \upsilon_{\eta }\left(\xi ,\phi ,\psi ,0\right)=\left(\cos(\xi )+\cos(\phi )+\cos(\psi )\right)-\left(\xi +\phi +\psi \right), \end{array}$$

with boundary conditions

(34) $$\begin{array}{cc} \; & \upsilon \left(0,\phi ,\psi ,\eta \right)=\left(-1+\phi +\psi -\cos(\phi )-\cos(\psi )\right)e^{-\eta },\\ \; & \upsilon \left(\frac{\pi }{3},\phi ,\psi ,\eta \right)=\left(\frac{2\pi -3}{6}+\phi +\psi -\cos\left(\phi \right)-\cos\left(\psi \right)\right)e^{-\eta },\\ \; & \upsilon \left(\xi ,0,\psi ,\eta \right)=\left(-1+\xi +\psi -\cos(\xi )-\cos(\psi )\right)e^{-\eta },\\ \; & \upsilon \left(\xi ,\frac{\pi }{3},\psi ,\eta \right)=\left(\frac{2\pi -3}{6}+\xi +\psi -\cos\left(\xi \right)-\cos\left(\psi \right)\right)e^{-\eta },\\ \; & \upsilon \left(\xi ,\phi ,0,\eta \right)=\left(-1+\xi +\phi -\cos(\xi )-\cos(\phi )\right)e^{-\eta },\\ \; & \upsilon \left(\xi ,\phi ,\frac{\pi }{3},\eta \right)=\left(\frac{2\pi -3}{6}+\xi +\phi -\cos\left(\xi \right)-\cos\left(\phi \right)\right)e^{-\eta },\\ \; & \upsilon_{\xi }\left(0,\phi ,\psi ,\eta \right)=\upsilon_{\phi }\left(\xi ,0,\psi ,\eta \right)=\upsilon_{\psi }\left(\xi ,\phi ,0,\eta \right)=e^{-\eta },\\ \; & \upsilon_{\xi }\left(\frac{\pi }{3},\phi ,\psi ,\eta \right)=\upsilon_{\phi }\left(\xi ,\frac{\pi }{3},\psi ,\eta \right)=\upsilon_{\psi }\left(\xi ,\phi ,\frac{\pi }{3},\eta \right)=\left(\frac{\sqrt{3}+2}{2}\right)e^{-\eta }. \end{array}$$

Concerning the natural transformation of (32), we get

$$\begin{array}{cc} & \upsilon (\xi ,\phi ,\psi ,s,u)=\frac{1}{s}\left\{\xi +\phi +\psi -\left(\cos(\xi )+\cos(\phi )+\cos(\psi )\right)\right\}+\frac{u}{s^{2}}\left\{\left(\cos(\xi )+\cos(\phi )+\cos(\psi )\right)-\left(\xi +\phi +\psi \right)\right\}\\ & -\frac{u(s-\gamma (s-u))}{s^{4}}N^{+}\left[2\left(\frac{\phi +\psi }{2\cos\xi }-1\right)\frac{\partial^{4}\upsilon }{\partial \xi^{4}}+2\left(\frac{\xi +\psi }{2\cos\phi }-1\right)\frac{\partial^{4}\upsilon }{\partial \phi^{4}}+2\left(\frac{\phi +\xi }{2\cos\psi }-1\right)\frac{\partial^{4}\upsilon }{\partial \psi^{4}}\right], \end{array}$$

using the inverse natural transform.

$$\begin{array}{cc} & \upsilon \left(\xi ,\phi ,\psi ,\eta \right)=N^{-}\left[\frac{1}{s}\left\{\xi +\phi +\psi -\left(\cos(\xi )+\cos(\phi )+\cos(\psi )\right)\right\}+\frac{u}{s^{2}}\left\{\left(\cos(\xi )+\cos(\phi )+\cos(\psi )\right)-\left(\xi +\phi +\psi \right)\right\}\right]\\ & -N^{-}\left[\frac{u(s-\gamma (s-u))}{s^{4}}N^{+}\left[2\left(\frac{\phi +\psi }{2\cos\xi }-1\right)\frac{\partial^{4}\upsilon }{\partial \xi^{4}}+2\left(\frac{\xi +\psi }{2\cos\phi }-1\right)\frac{\partial^{4}\upsilon }{\partial \phi^{4}}+2\left(\frac{\phi +\xi }{2\cos\psi }-1\right)\frac{\partial^{4}\upsilon }{\partial \psi^{4}}\right]\right], \end{array}$$

(35) $$\begin{array}{cc} & \upsilon \left(\xi ,\phi ,\psi ,\eta \right)=\left\{\xi +\phi +\psi -\left(\cos(\xi )+\cos(\phi )+\cos(\psi )\right)\right\}\left(1-\eta \right)-N^{-}\left[\frac{u(s-\gamma (s-u))}{s^{4}}N^{+}\left\{2\left(\frac{\phi +\psi }{2\cos\xi }-1\right)\frac{\partial^{4}\upsilon }{\partial \xi^{4}}\right.\right.\\ & \left.\left.+2\left(\frac{\xi +\psi }{2\cos\phi }-1\right)\frac{\partial^{4}\upsilon }{\partial \phi^{4}}+2\left(\frac{\phi +\xi }{2\cos\psi }-1\right)\frac{\partial^{4}\upsilon }{\partial \psi^{4}}\right\}\right], \end{array}$$

The Equation (35) correction function is provided by

(36) $$\begin{array}{cc} \; & \sum_{\ell =0}^{\infty }\upsilon_{\ell +1}\left(\xi ,\phi ,\eta \right)=\left\{\xi +\phi +\psi -\left(\cos(\xi )+\cos(\phi )+\cos(\psi )\right)\right\}\left(1-\eta \right)-N^{-}\left[\frac{u(s-\gamma (s-u))}{s^{4}}\right.\\ \; & \left.N^{+}\left\{2\left(\frac{\phi +\psi }{2\cos\xi }-1\right)\sum_{\ell =0}^{\infty }\frac{\partial^{4}\upsilon_{\ell }}{\partial \xi^{4}}+2\left(\frac{\xi +\psi }{2\cos\phi }-1\right)\sum_{\ell =0}^{\infty }\frac{\partial^{4}\upsilon_{\ell }}{\partial \phi^{4}}+2\left(\frac{\phi +\xi }{2\cos\psi }-1\right)\sum_{\ell =0}^{\infty }\frac{\partial^{4}\upsilon_{\ell }}{\partial \psi^{4}}\right\}\right], \end{array}$$

The first term being

(37) $$\upsilon_{0}\left(\xi ,\phi ,\psi ,\eta \right)=\left\{\xi +\phi +\psi -\left(\cos(\xi )+\cos(\phi )+\cos(\psi )\right)\right\}\left(1-\eta \right),$$

Then we get

(38) $$\begin{array}{cc} \upsilon_{\ell +1}\left(\xi ,\phi ,\psi ,\eta \right)= & -N^{-}\left[\frac{u(s-\gamma (s-u))}{s^{4}}N^{+}\left\{2\left(\frac{\phi +\psi }{2\cos\xi }-1\right)\sum_{\ell =0}^{\infty }\frac{\partial^{4}\upsilon_{\ell }}{\partial \xi^{4}}\right.\right.\\ \; & \left.\left.+2\left(\frac{\xi +\psi }{2\cos\phi }-1\right)\sum_{\ell =0}^{\infty }\frac{\partial^{4}\upsilon_{\ell }}{\partial \phi^{4}}+2\left(\frac{\phi +\xi }{2\cos\psi }-1\right)\sum_{\ell =0}^{\infty }\frac{\partial^{4}\upsilon_{\ell }}{\partial \psi^{4}}\right\}\right], \end{array}$$

for j=0

(39) $$\begin{array}{cc} & \upsilon_{1}\left(\xi ,\phi ,\psi ,\eta \right)=-N^{-}\left[\frac{u(s-\gamma (s-u))}{s^{4}}N^{+}\left\{2\left(\frac{\phi +\psi }{2\cos\xi }-1\right)\frac{\partial^{4}\upsilon_{0}}{\partial \xi^{4}}+2\left(\frac{\xi +\psi }{2\cos\phi }-1\right)\frac{\partial^{4}\upsilon_{0}}{\partial \phi^{4}}+2\left(\frac{\phi +\xi }{2\cos\psi }-1\right)\frac{\partial^{4}\upsilon_{0}}{\partial \psi^{4}}\right\}\right],\\ & \upsilon_{1}\left(\xi ,\phi ,\psi ,\eta \right)=\left\{\xi +\phi +\psi -\left(\cos(\xi )+\cos(\phi )+\cos(\psi )\right)\right\}\left(\left(1-\gamma +\gamma \eta \right)-\frac{\eta^{2}}{3!}\left(3-3\gamma +\gamma \eta \right)\right). \end{array}$$

The following terms are

(40) $$\begin{array}{cc} & \upsilon_{2}\left(\xi ,\phi ,\psi ,\eta \right)=-N^{-}\left[\frac{u(s-\gamma (s-u))}{s^{4}}N^{+}\left\{2\left(\frac{\phi +\psi }{2\cos\xi }-1\right)\frac{\partial^{4}\upsilon_{1}}{\partial \xi^{4}}+2\left(\frac{\xi +\psi }{2\cos\phi }-1\right)\frac{\partial^{4}\upsilon_{1}}{\partial \phi^{4}}+2\left(\frac{\phi +\xi }{2\cos\psi }-1\right)\frac{\partial^{4}\upsilon_{1}}{\partial \psi^{4}}\right\}\right],\\ & \upsilon_{2}\left(\xi ,\phi ,\psi ,\eta \right)=\left\{\xi +\phi +\psi -\left(\cos(\xi )+\cos(\phi )+\cos(\psi )\right)\right\}\left(\frac{\eta^{3}}{4!}\left(4-4\gamma +\gamma \eta \right)-\frac{\eta^{4}}{5!}\left(\gamma \eta +5-5\gamma \right)\right),\\ & \upsilon_{3}\left(\xi ,\phi ,\psi ,\eta \right)=N^{-}\left[\frac{u(s-\gamma (s-u))}{s^{4}}N^{+}\left\{2\left(\frac{\phi +\psi }{2\cos\xi }-1\right)\frac{\partial^{4}\upsilon_{2}}{\partial \xi^{4}}+2\left(\frac{\xi +\psi }{2\cos\phi }-1\right)\frac{\partial^{4}\upsilon_{2}}{\partial \phi^{4}}+2\left(\frac{\phi +\xi }{2\cos\psi }-1\right)\frac{\partial^{4}\upsilon_{2}}{\partial \psi^{4}}\right\}\right],\\ & \upsilon_{3}\left(\xi ,\phi ,\psi ,\eta \right)=\left\{\xi +\phi +\psi -\left(\cos(\xi )+\cos(\phi )+\cos(\psi )\right)\right\}\left(\frac{\eta^{5}}{6!}\left(6-6\gamma +\gamma \eta \right)-\frac{\eta^{6}}{7!}\left(\gamma \eta +7-7\gamma \right)\right)\cdots . \end{array}$$

The series forms a solution to Problems (4.3), for example,

$$\upsilon \left(\xi ,\phi ,\psi ,\eta \right)=\upsilon_{0}\left(\xi ,\phi ,\psi ,\eta \right)+\upsilon_{1}\left(\xi ,\phi ,\psi ,\eta \right)+\upsilon_{2}\left(\xi ,\phi ,\psi ,\eta \right)+\upsilon_{3}\left(\xi ,\phi ,\psi ,\eta \right)+\upsilon_{4}\left(\xi ,\phi ,\psi ,\eta \right)\cdots .$$

$$\begin{array}{cc} & \upsilon \left(\xi ,\phi ,\psi ,\eta \right)=\left\{\xi +\phi +\psi -\left(\cos(\xi )+\cos(\phi )+\cos(\psi )\right)\right\}\{1-\eta +\left(1-\gamma +\gamma \eta \right)-\frac{\eta^{2}}{3!}\left(3-3\gamma +\gamma \eta \right)\\ & +\frac{\eta^{3}}{4!}\left(4-4\gamma +\gamma \eta \right)-\frac{\eta^{4}}{5!}\left(\gamma \eta +5-5\gamma \right)+\frac{\eta^{5}}{6!}\left(6-6\gamma +\gamma \eta \right)-\frac{\eta^{6}}{7!}\left(\gamma \eta +7-7\gamma \right)\cdots \}. \end{array}$$

Then γ=1; the integer NDM results as

(41) $$\begin{array}{cc} \; & \upsilon \left(\xi ,\phi ,\psi ,\eta \right)=\left\{\xi +\phi +\psi -\left(\cos(\xi )+\cos(\phi )+\cos(\psi )\right)\right\}\{1-\eta +\frac{\eta^{2}}{2!}-\frac{\eta^{3}}{3!}\\ \; & +\frac{\eta^{4}}{4!}-\frac{\eta^{5}}{5!}+\frac{\eta^{6}}{6!}-\frac{\eta^{7}}{7!}\cdots \}. \end{array}$$

The exact solution is

$$\upsilon \left(\xi ,\phi ,\psi ,\eta \right)=\left(\xi +\phi +\psi -\left(\cos(\xi )+\cos(\phi )+\cos(\psi )\right)\right)e^{-\eta }.$$

**Figure 5:**
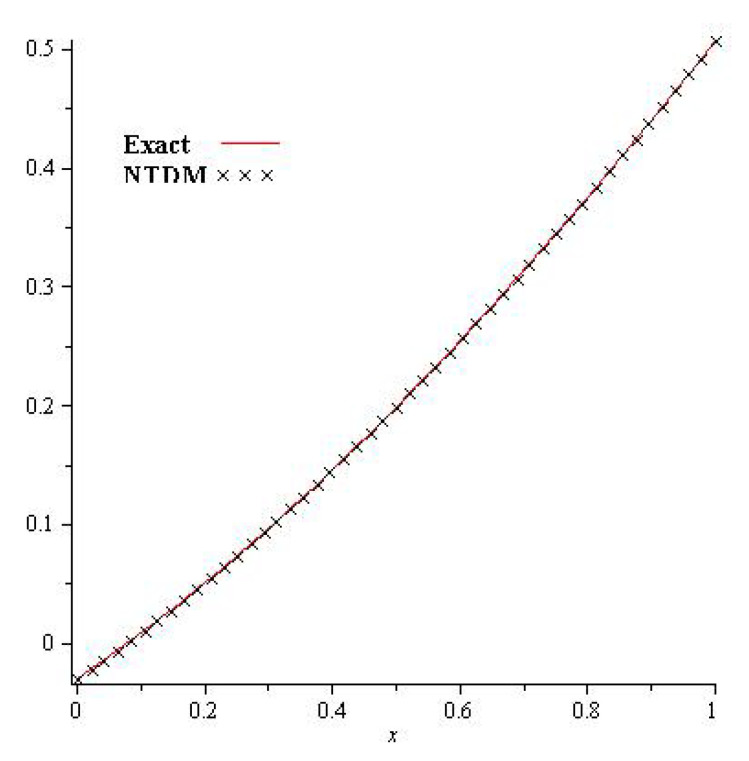
The exact and NTDM solution for γ=1 of Problem 4.3.

**Figure 6:**
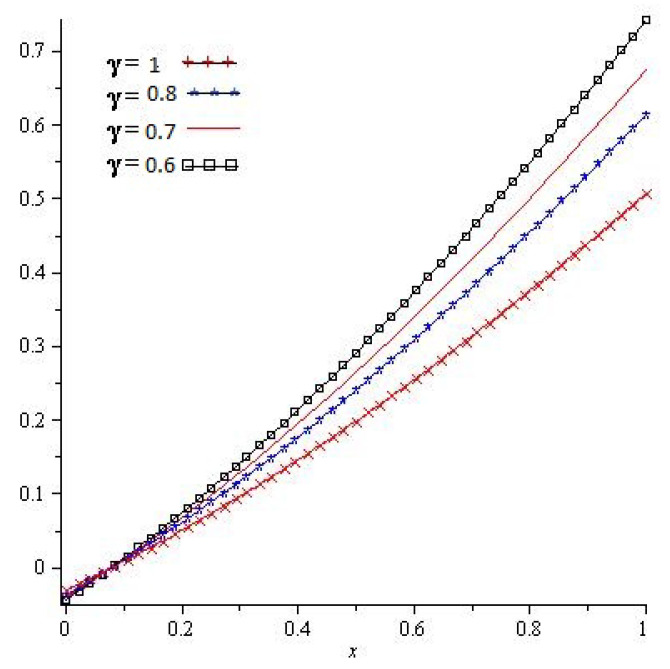
For different values of γ of Problem 4.3.

Figure 5, show that the exact and analytical solution graph of Problem 4.3. In Figure 6, the obtained solutions of Problem 4.3 are plotted at various fractional orders of the derivatives; it is confirmed that the exact and derived results are in close contact with each other. Thus, the proposed method provided an accurate solution for Problem 4.3.

## 5. Conclusions

In the present article, an efficient analytical technique is used to solve fractional-order parabolic equations. The present method is the combinations of two well-known methods, namely the natural transform and Adomian decomposition method. The natural transform is applied to the given problem, which makes it easier. After this, we implemented the Adomian decomposition method and then the inverse natural transform to get the closed form analytical solutions for the given problems. The proposed method requires a small number of calculation to attain closed form solutions and is therefore considered to be one of the best analytical techniques to solve fractional-order partial differential equations.

## Data Availability

Not applicable.

## References

[1] Khaliq A.Q.M., Twizell E.H. (1987). A family of second order methods for variable coefficient fourth order parabolic partial differential equations. Int. J. Comput. Math..

[2] Gorman D.J. (1975). Free Vibration Analysisi of Beams and Shafts(Book).

[3] Andrade C., McKee S. (1977). High accuracy ADI methods for fourth order parabolic equations with variable coefficients. J. Comput. Appl. Math..

[4] Conte S.D. (1957). A stable implicit finite difference approximation to a fourth order parabolic equation. J. ACM.

[5] Royster W.C., Conte S.D. (1956). Convergence of finite difference solutions to a solution of the equation of the vibrating rod. Proc. Am. Math. Soc..

[6] Evans D.J. (1965). A stable explicit method for the finite-difference solution of a fourth-order parabolic partial differential equation. Comput. J..

[7] Evans D.J., Yousif W.S. (1991). A note on solving the fourth order parabolic equation by the AGE method. Int. J. Comput. Math..

[8] Wazwaz A.-M. (1995). On the solution of the fourth order parabolic equation by the decomposition method. Int. J. Comput. Math..

[9] Liao W., Zhu J., Khaliq A.Q.M. (2002). An efficient high-order algorithm for solving systems of reaction-diffusion equations. Numer. Methods Part. Differ. Equ..

[10] Jain M.K., Iyengar S.R.K., Lone A.G. (1976). Higher order difference formulas for a fourth order parabolic partial differential equation. Int. J. Numer. Methods Eng..

[11] Caglar H., Caglar N. (2008). Fifth-degree B-spline solution for a fourth-order parabolic partial differential equations. Appl. Math. Comput..

[12] Wazwaz A.-M. (2001). Analytic treatment for variable coefficient fourth-order parabolic partial differential equations. Appl. Math. Comput..

[13] Rashidinia J., Mohammadi R. (2010). Sextic spline solution of variable coefficient fourth-order parabolic equations. Int. J. Comput. Math..

[14] Aziz T., Khan A., Rashidinia J. (2005). Spline methods for the solution of fourth-order parabolic partial differential equations. Appl. Math. Comput..

[15] Biazar J., Ghazvini H. (2007). Hes variational iteration method for fourth-order parabolic equations. Comput. Math. Appl..

[16] Noor M.A., Noor K.I., Mohyud-Din S.T. (2009). Modified variational iteration technique for solving singular fourth-order parabolic partial differential equations. Nonlinear Anal. Theory Methods Appl..

[17] Dehghan M., Manafian J. (2009). The solution of the variable coefficients fourth-order parabolic partial differential equations by the homotopy perturbation method. Z. Naturforschung A.

[18] Nadeem M., Li F., Ahmad H. (2019). Modified Laplace variational iteration method for solving fourth-order parabolic partial differential equation with variable coefficients. Comput. Math. Appl..

[19] Rawashdeh M., Maitama S. (2017). Finding exact solutions of nonlinear PDEs using the natural decomposition method. Math. Methods Appl. Sci..

[20] Baskonus H.M., Bulut H., Pandir Y. (2014). The natural transform decomposition method for linear and nonlinear partial differential equations. Math. Eng. Sci. Aerosp. (MESA).

[21] Adomian G. (1984). A new approach to nonlinear partial differential equations. J. Math. Anal. Appl..

[22] Belgacem F.B.M., Silambarasan R. (2012). Theory of natural transform. J. MESA.

[23] Khan H., Shah R., Kumam P., Arif M. (2019). Analytical Solutions of Fractional-Order Heat and Wave Equations by the Natural Transform Decomposition Method. Entropy.

[24] Shah R., Khan H., Kumam P., Arif M., Baleanu D. (2019). Natural Transform Decomposition Method for Solving Fractional-Order Partial Differential Equations with Proportional Delay. Mathematics.

[25] Shah R., Khan H., Mustafa S., Kumam P., Arif M. (2019). Analytical Solutions of Fractional-Order Diffusion Equations by Natural Transform Decomposition Method. Entropy.

[26] Rawashdeh M.S., Al-Jammal H. (2016). New approximate solutions to fractional nonlinear systems of partial differential equations using the FNDM. Adv. Differ. Equ..

[27] Eltayeb H., Abdalla Y.T., Bachar I., Khabir M.H. (2019). Fractional telegraph equation and its solution by natural transform decomposition method. Symmetry.

[28] Prakasha D.G., Veeresha P., Rawashdeh M.S. (2019). Numerical solution for (2+1)-dimensional time-fractional coupled Burger equations using fractional natural decomposition method. Math. Methods Appl. Sci..

[29] Belgacem F.B.M., Silambarasan R. (2012). Advances in the natural transform. AIP Conf. Proc..

[30] Khan Z.H., Khan W.A. (2008). N-transform properties and applications. NUST J. Eng. Sci..

[31] Hilfer R. (2000). Applications of Fractional Calculus in Physics.

[32] Podlubny I. (1998). Fractional Differential Equations: An Introduction to Fractional Derivatives, Fractional Differential Equations, to Methods of Their Solution and Some of Their Applications.

[33] Miller K.S., Ross B. (1993). An Introduction to the Fractional Calculus and Fractional Differential Equations.

